# Fluorescence DNA Switch for Highly Sensitive Detection of miRNA Amplified by Duplex-Specific Nuclease

**DOI:** 10.3390/s22093252

**Published:** 2022-04-23

**Authors:** Xiaoqiang Li, Zhenzhen Guo, Gangyin Luo, Peng Miao

**Affiliations:** 1 School of Biomedical Engineering (Suzhou), University of Science and Technology of China, Hefei 230026, China; lixq@sibet.ac.cn; 2 Suzhou Institute of Biomedical Engineering and Technology, Chinese Academy of Sciences, Suzhou 215163, China; guozz@sibet.ac.cn (Z.G.); luogy@sibet.ac.cn (G.L.); 3 Ji Hua Laboratory, Foshan 528200, China

**Keywords:** miRNA, fluorescent sensor, duplex-specific nuclease, gold nanoparticles, DNA switch

## Abstract

DNA is a type of promising material for the construction of sensors owing to its sequence programmability to control the formation of certain structures. MicroRNA (miRNA) can be applied as promising biomarkers for the diagnosis of a range of diseases. Herein, a novel fluorescent sensing strategy for miRNA is proposed combining duplex-specific nuclease (DSN)-mediated amplification and dumbbell DNA structural switch. Gold nanoparticles (AuNPs) are employed, which provide a 3D reaction interface. They also act as effective fluorescence quenchers. The proposed sensor exhibits high sensitivity (sub-femtomolar level) with a wide dynamic range. In addition, excellent selectivity to distinguish homology sequences is achieved. It also performs satisfactorily in biological samples. Overall, this fluorescent sensor provides a powerful tool for the analysis of miRNA levels and can be applied for related biological studies and clinical diagnosis.

## 1. Introduction

MicroRNA (miRNA) is a type of small noncoding RNAs, which plays a critical regulatory role in gene expression by binding with protein coding mRNA [1,2]. MiRNAs have important functions in several biological events including cell proliferation, differentiation and apoptosis [3,4]. They are currently regarded as a kind of biomarker candidate for the diagnosis and prognosis of diseases [5,6]. A number of miRNAs have been found to have irregular expression levels in pathological states [7], which have inspired considerable work on miRNA profiling for different types of cancers over the past few decades [8,9]. Currently, different strategies have been developed, including the most commonly applied quantitative real-time polymerase chain reaction (qRT-PCR), microarray, gel assay and sequencing techniques [10,11,12]. Various sensors have also been developed for sensitive and selective analysis of miRNA, such as fluorescent, electrochemical, and electrogenerated chemiluminescent sensors [13,14,15,16]. DNA or RNA structural transitions have been applied as the basis for target recognition and detection [17,18]. For example, Aw et al. introduced an additional stem loop into fluorescent RNA spinach and altered its 3′ and 5′ terminals. A new RNA named Pandan was generated, which encoded the complementary sequence of a target. Target miRNA could be conjugated, leading to the reconstitution of RNA scaffold for the binding of fluorophore and signal output [19]. Tavallaie et al. fabricated a sensitive method for miRNA detection based on the use of DNA modified gold/magnetic composites [20]. Electric-field-induced dynamic assembly of the nanoconjugate network provided a precise and facile way to directly analyze target miRNA (from 10 aM to 1 nM) in unprocessed blood samples. Our group developed a novel DNA walking and rolling nanomachine for electrochemical detection of miRNA on the basis of DNA modified gold nanoparticles (AuNPs) and duplex-specific nuclease (DSN) mediated amplification [21]. Single-stranded DNA or RNA are resistant to DSN. It only digests double-stranded DNA or DNA in DNA/RNA heteroduplexes. Due to this unique enzyme activity, DSN has become a popular tool for RNA analysis [22,23].

Novel sensors show great potential for fast and accurate determination of miRNA. However, more efforts should be made in order to further improve the analytical performances, as well as anti-interference capability in biological samples. In this contribution, we develop a novel fluorescent sensor for miRNA based on a DNA structural switch and DSN-mediated amplification. In addition, AuNPs are utilized, which not only provide a 3D reaction interface, but also act as fluorescence quenchers [24]. The developed sensor shows excellent sensitivity and selectivity. Good practical utility is also confirmed by introducing serum sample tests. Therefore, the proposed miRNA sensor holds great potential use for biochemical and biomedical applications.

## 2. Materials and Methods

### 2.1. Materials and Chemicals

Gold(III) chloride trihydrate (HAuCl_4_·3H_2_O), ethylenediaminetetraacetic acid (EDTA), diethypyrocarbonate (DEPC), and tris(2-carboxyethyl)phosphine hydrochloride (TCEP) were purchased from Sigma (USA). DSN was purchased from Genomax Technologies Pte Ltd. (Singapore). All the other chemicals were of analytical grade and used as received. All oligonucleotides were synthesized and purified by Sangon Biotech Co., Ltd. (Shanghai, China). The sequences were listed in Table 1. All strands were dissolved in 10 mM Tris-HCl buffer (1 mM EDTA, 10 mM TCEP, and 0.1 M NaCl, pH 7.4) with a concentration of 100 μM.

### 2.2. Preparation of DNA Modified AuNPs

Bare AuNPs were synthesized by means of citrate reduction of HAuCl_4_, according to our previous report [25]. Generally, HAuCl_4_ and trisodium citrate solutions were prepared with the concentrations of 0.01% (*w*/*v*) and 1% (*w*/*v*), separately. Then, 3.5 mL of trisodium citrate was added to 100 mL of refluxing HAuCl_4_ solution under stirring and boiling for 15 min. Subsequently, the heat was removed and the mixture was stirred for another 30 min. Next, the solution was cooled down to room temperature. The synthesized AuNPs were purified by three cycles of centrifugation at 12,000 rpm for 20 min (Eppendorf rotor: FA-45-30-11).

To achieve the attachment of the thiolated DNA Probe A on the surface of AuNPs, a pH-assisted route was utilized [26]. Briefly, Probe A was directly added to AuNPs with the concentration of 4 μM. The final volume was 1 mL. After incubation for 2 min, pH was adjusted to 3.0 with the use of citrate buffer. Unreacted Probe A strands could be removed by centrifuging at 12,000 rpm for 20 min. The precipitates were resuspended in 10 mM phosphate buffered saline (PBS).

### 2.3. Preparation of Gold Electrode

Substrate gold electrode (2 mm diameter) was firstly treated with piranha solution (98% H_2_SO_4_: 30% H_2_O_2_ = 3:1) for 5 min (*Caution: Highly dangerous*). After carefully rinsing with double-distilled water, the electrode was polished with P5000 silicon carbide paper and then alumina slurry (1, 0.3, 0.05 μm), respectively. The electrode was further sonicated in ethanol and pure water. Next, it was electrochemically cleaned with 0.5 M H_2_SO_4_. After it was dried with nitrogen, the electrode was ready for further modification and measurements.

### 2.4. DSN-Mediated Amplification and Dumbbell DNA Switch

A series of standard miRNA solutions (10^−16^, 10^−15^, 10^−14^, 10^−13^, 10^−12^, 10^−11^, 10^−10^, 10^−9^, 10^−8^, 2 × 10^−8^) were prepared, which were then incubated with Probe A modified AuNPs in the presence of 1.0 unit of DSN. The solutions were maintained at 50 °C (water bath) for 120 min. After that, centrifugations were performed and the supernatants were collected, which were further mixed with Probe C (400 nM). After incubating for another 20 min at room temperature, bare AuNPs with the same volumes were added. 0.8 mL of the solutions were then transferred into the cuvette for fluorescent measurements. 

### 2.5. Electrochemical Measurements

An electrochemical analyzer (CHI660D, CH Instrument, Shanghai, China) was used to conduct electrochemical experiments. A three-electrode system was employed consisting of the gold working electrode, the platinum auxiliary electrode and the saturated calomel reference electrode. Electrochemical impedance spectroscopy (EIS) was carried out in 5 mM [Fe(CN)_6_]^3−/4−^ with 1 M KCl. The parameters included 0.23 V biasing potential, 5 mV amplitude, 0.1 Hz to 100 kHz frequency range. 

### 2.6. Fluorescent Measurements

All fluorescence spectra were measured using an F-4600 Fluorescence Spectrophotometer (Hitachi, Japan). The excitation wavelength of 488 nm and the scan range of 500 to 650 nm were applied. The parameters of quartz cuvette include optical path, 10 mm; volume, 3.5 mL; light transmittance, over 80%; size, 12.5 mm × 12.5 mm × 45 mm.

### 2.7. UV-vis Measurements and Polyacrylamide Gel Electrophoresis (PAGE)

UV-vis absorbance spectra of AuNPs before and after certain treatments were performed using a NanoDrop OneC spectrophotometer (Thermo Scientific, USA). The spectra were recorded from 225 to 800 nm. PAGE experiments were performed in the Tris-boric acid buffer solution (90 mM, 1 mM EDTA, pH 8.0) at 120 V for 45 min. Next, the polyacrylamide gel (10%) was stained with 4S Red Plus solution, which was then photographed under UV light by the Gel DocTM XR+ Imaging System (Bio-Rad, USA).

## 3. Results and Discussion

### 3.1. Sensing Principle

**Scheme 1:**
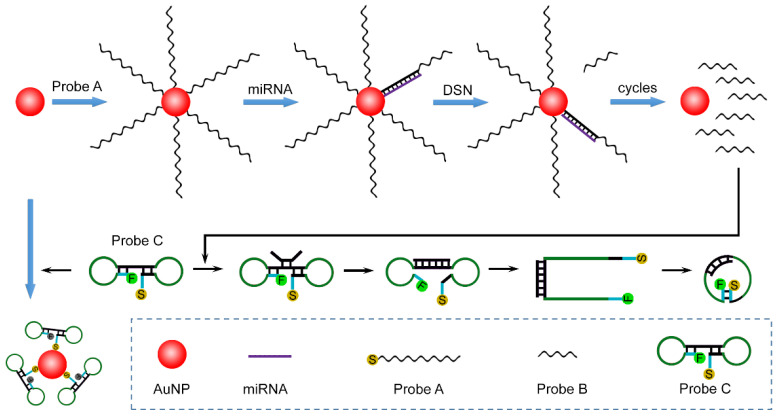
Illustration of the sensing strategy for the detection of miRNA (DSN: duplex-specific nuclease; AuNP: gold nanoparticle; F: Alexa Fluor 488; S: thiol group).

The working mechanism of the proposed sensor is illustrated in Scheme 1; hsa-let-7a is applied as the example of target miRNA. The process can be divided into two steps. First, bare AuNPs are functionalized with Probe A via gold-sulfur chemistry. In the presence of miRNA, a hybridization event occurs between Probe A and miRNA. The formed DNA/RNA duplex can be recognized by DSN and the DNA strand can be digested, releasing the other single-stranded segment (Probe B) and miRNA. Target miRNA is recycled to generate a larger number of Probe B strands. Second, dumbbell-structured Probe C is designed with 3′ terminal Alexa Fluor 488 and 5′ terminal thiol group. In the original configuration, the 5′ terminal is exposed, facilitating the interaction between the thiol group and AuNPs. The fluorescence reporter could thus be quenched by AuNPs and reflects nearly no emission. Nevertheless, in the presence of Probe B strands, the stem part of Probe C interacts with Probe B sequence and the two loops are relieved. In addition, self-hybridization is achieved between the 5′ and 3′ terminals. As a result, the thiol group is hidden in the new stem segment, inhibiting the adsorption of Probe C on AuNPs. Without Förster resonance energy transfer (FRET), Alexa Fluor 488 exhibits strong emission intensity, which could be applied to indicate the initial concentration of miRNA. 

### 3.2. Feasibility of the Working Mechanism

**Figure 1:**
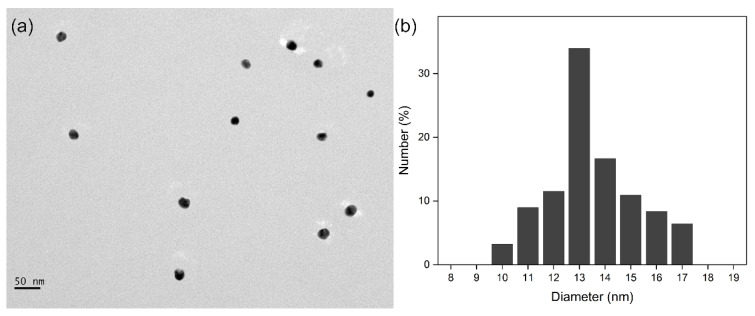
(**a**) Transmission electron microscopic image of AuNPs. (**b**) Size distribution of AuNPs.

**Figure 2:**
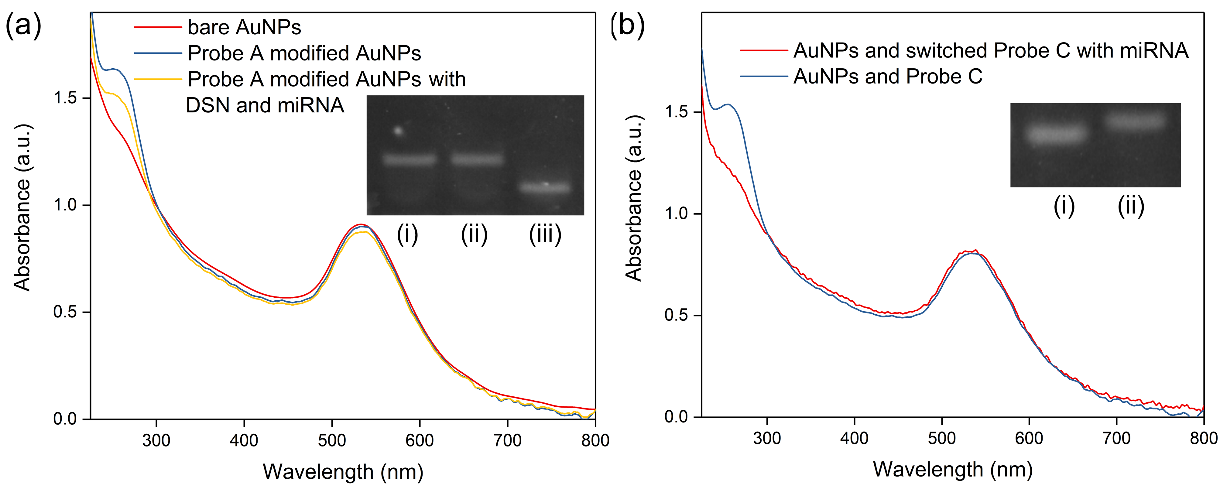
(**a**) UV−vis absorption spectra of bare AuNPs and Probe A modified AuNPs before and after treated with DSN in the presence of target miRNA. Inset is the PAGE image of (i) Probe A, (ii) Probe A after DSN treatment, and (iii) Probe A after DSN treatment in the presence of miRNA. (**b**) UV−vis absorption spectra of bare AuNPs treated with Probe C before and after the miRNA-mediated structural switch. Inset is the PAGE image of (i) Probe C, and (ii) the mixture of Probe B and C.

The diameter of the synthesized AuNPs is about 13 nm, the morphology of which show sphere structures. AuNPs are also well dispersed in the solution (Figure 1). The DSN catalyzed reaction and the Probe B mediated dumbbell DNA transition reaction are then characterized. As shown in Figure 2a, Probe A modified AuNPs reflect two UV-vis absorbance peaks. The one at 520 nm is ascribed to the localized surface plasmon resonance absorbance (LSPR) of AuNPs. The other at 260 nm is due to the existence of DNA bases. After treatment with DSN in the presence of a target, the peak at 260 decreases, confirming the DSN catalyzed reaction. More evidence can be directly verified by the PAGE image in the inset. After treatment with DSN directly, the band of Probe A (lane ii) is nearly the same as the pure strand (lane i). With miRNA induced formation of the DNA/RNA duplex, it could be cleaved and the shortened DNA runs faster (lane iii). On the other hand, significant peaks at 260 nm and 520 nm can also be found in the UV-vis absorption spectrum of Probe C modified AuNPs. However, with Probe B strands generated in the presence of the target, Probe C undergoes a structural switch, which can no longer be attached on the surface of AuNPs. Therefore, after centrifugation, the 260 nm peak disappears in the resuspended solution (Figure 2b). In the PAGE image, the formed Probe B/C structure (lane ii) runs slower in the gel compared with dumbbell structured Probe C (lane i), demonstrating a successful DNA structural switch.

**Figure 3:**
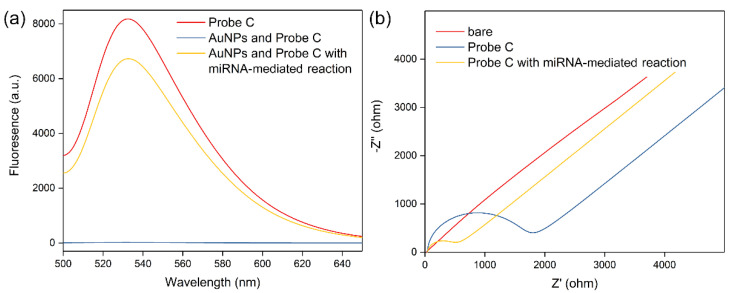
(**a**) Fluorescence emission spectra of Probe C, AuNPs and Probe C before and after miRNA-mediated structural switch. (**b**) Nyquist plots of the bare gold electrode and electrode modified with Probe C without and with structural switch.

The reaction process is then characterized by fluorescent and electrochemical techniques. As shown in Figure 3a, a significant fluorescence peak around 530 nm is observed in Probe C solution due to the labeled Alexa Fluor 488. After mixing Probe C and AuNPs, the strands could be attached on the surface of AuNPs. The proximity of a metal nanoparticle to the organic fluorophore quenches the fluorescence [27]. However, after the structural switch of Probe C, the thiol group is hidden. The conjugation of Probe C and AuNPs is thus inhibited. Therefore, the quenching of Alexa Fluor 488 fluorescence cannot be achieved. We have further investigated the structural switch on the electrodes by EIS. In a typical nyquist plot, the diameter of a semicircle domain reflects the charge transfer resistance of the modified layer on the electrode. As shown in Figure 3b, after the incubation of the bare electrode with Probe C directly, a larger semicircle domain is observed, which is due to the repellant between modified DNA strands and electrochemical species. However, miRNA induced reactions can be applied to assist the structural switch of Probe C. With the hidden thiol group, Probe C cannot be effectively immobilized on the surface of the electrode, leading to the significant decrease of charge transfer resistance. There results well confirm the feasibility of the sensing mechanism.

### 3.3. Quantification of miRNA

**Figure 4:**
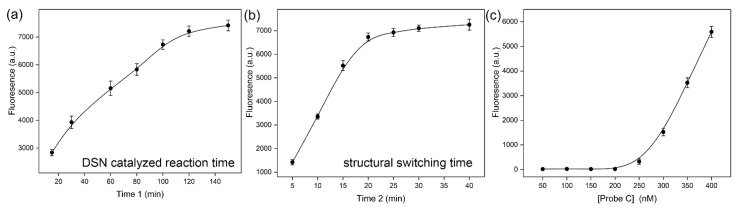
Optimization of (**a**) DSN catalyzed reaction time, (**b**) structural switching time, (**c**) Probe C concentration. MiRNA concentration of 1 nM and Probe C concentration of 200 nM are used for the optimization of reaction times.

To achieve better analytical performances, several important parameters were first optimized. With longer times of DSN catalyzed reaction and Probe B assisted structural transition of Probe C, more significant fluorescence responses could be obtained. By comparing the increasing trends of fluorescence with reaction times, 120 min and 20 min are selected as optimal values (Figure 4a,b). The concentration of Probe C (labeled with Alexa Fluor 488) is also important to the finally recorded fluorescence intensity; a lesser amount of Probe C might be adsorbed on AuNPs and fluorescence is completely quenched. With the increase of Probe C, the sites on the surface of AuNPs might be occupied gradually and free Probe C in the solution emit stronger fluorescence. As shown in Figure 4c, with the concentration of Probe C lower than 200 nM, the fluorescence is still quenched. However, more Probe C strands might lead to the gradual increase of Alexa Fluor 488 signal. Therefore, 200 nM Probe C is applied in the following experiments.

**Figure 5:**
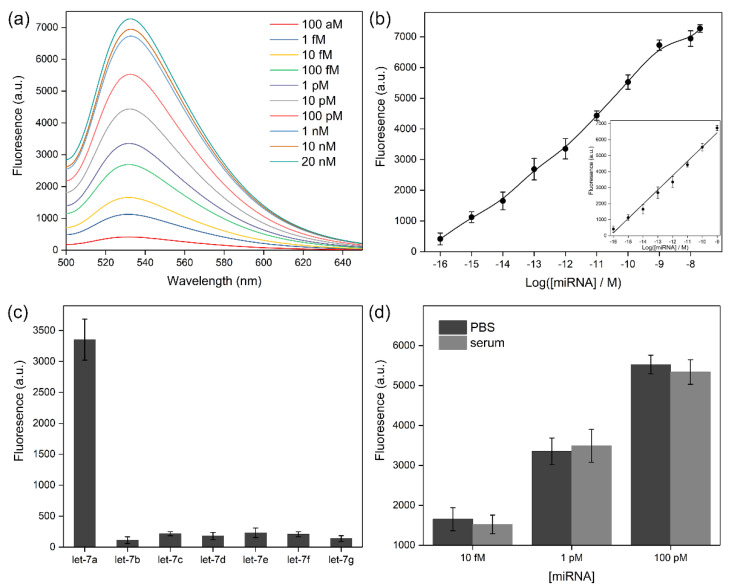
(**a**) Fluorescence emission spectra for the detection of miRNA with different concentrations. (**b**) Calibration curve representing the relationship between peak intensity and miRNA concentration. Inset is the linear range. Error bars represent standard deviations of three independent measurements. (**c**) Selectivity investigation of target and homology miRNAs. (**d**) Fluorescence performances of the sensor in PBS buffer and serum samples spiked with different amounts of miRNA.

Under the optimized conditions, the detection of miRNA with a series of concentrations (10^−16^, 10^−15^, 10^−14^, 10^−13^, 10^−12^, 10^−11^, 10^−10^, 10^−9^, 10^−8^, 2 × 10^−8^) are carried out, and corresponding fluorescence emission spectra are recorded in Figure 5a. With the increase of target miRNA, the fluorescence peak increases accordingly. The detailed relationship between the peak intensity and the logarithmic concentration of miRNA is depicted in Figure 5b. A linear range is established from 10^−16^ to 10^−9^ M, which is rather wide. The equation is as follows:

*y* = 14467.60 + 892.94 *x* (*n* = 3, *R*^2^ = 0.99) (1)

in which *y* stands for the fluorescence peak intensity, and *x* is logarithmic concentration of miRNA. The limit of detection is calculated to be 10^−16^ M (S/N = 3). Compared with recently reported sensors, the performances are satisfactory and show certain superiority (Table 2). The operation is facile and the reaction system is simple. Due to the amplification of DSN catalyzed reaction, trace miRNA levels can be easily evaluated. Although qPT-PCR shows a lower limit of detection, this sensor is sufficiently sensitive for the analysis of miRNA in biological samples.

### 3.4. Investigations of Selectivity and Practical Utility

After demonstrating the sensing performance of this strategy, several potential interfering miRNAs were introduced in the buffer solutions to check the selectivity of the proposed method. As shown in Figure 5c, miRNAs of the let-7 family are introduced in the system, and the finally recorded fluorescence peak intensities are compared. It is clearly shown that only let-7a could initiate the reactions as expected, which induce the increase of the fluorescence peak. Target miRNA could be easily distinguished from other negative controls. We have further spiked different amounts of target miRNA in serum samples, which were then tested by the proposed method. With the increase of miRNA concentration, the fluorescence response increased correspondingly. In addition, the detailed values are in good accordance with the performances in PBS conditions (Figure 5d). These results confirm the feasibility of this method in biological samples. 

## 4. Conclusions

In summary, a novel fluorescent sensor for highly sensitive detection of miRNA is developed, combining the DSN-mediated digestion reaction and a DNA structural switch. Owing to the large surface area of AuNPs that could load multiple Probe A strands for the DSN catalyzed reaction, abundant Probe B strands are thus produced to initiate the structural change of Probe C, leading to the abolishment of FRET from the fluorescence probe to AuNPs. The recorded signal could be a good reflection of the initiator miRNA. This proposed sensor allows the detection of miRNA down to 0.1 fM, with good reproducibility. It also discriminates interfering miRNAs with excellent selectivity. The developed sensing strategy offers a powerful tool for biomedical research, and can be utilized for the applications of cancer diagnosis.

## Figures and Tables

**Table 1:** DNA and RNA sequences.

Name	Sequence (from 5′ to 3′)
Probe A	GTTGGAGCTAGTGGCGTAGAACTATACAACCTACTACCTCA-(CH_2_)_6_-SH
Probe B	GTTGGAGCTAGTGGCGTAG
Probe C	SH-(CH_2_)_3_-TCCAACGGCGTAGTCGAAATAGGCTACGCCACTAGCTCCAACTCGAAATAGGGTTGGATTT-Alexa Fluor 488
hsa-let-7a	UGAGGUAGUAGGUUGUAUAGUU
hsa-let-7b	UGAGGUAGUAGGUUGUGUGGU
hsa-let-7c	UGAGGUAGUAGGUUGUAUGGUU
hsa-let-7d	AGAGGUAGUAGGUUGCAUAGUU
hsa-let-7e	UGAGGUAGGAGGUUGUAUAGUU
hsa-let-7f	UGAGGUAGUAGAUUGUAUAGUU
hsa-let-7g	UGAGGUAGUAGUUUGUACAGUU

**Table 2:** Comparison of the analytical performances of recent miRNA assays.

Technique	Strategy	LOD (M)	Ref
fluorescence	self-assembled DNA dendrimers	2.5 × 10^−^^9^	[28]
fluorescence	light-up RNA aptamers	3 × 10^−^^10^	[29]
surface-enhanced Raman scattering	target responsive DNA hydrogel	1.1 × 10^−^^10^	[30]
fluorescence	catalyzed hairpin assembly based ratiometric assay	7.2 × 10^−^^11^	[31]
photoelectrochemistry	dual cascade toehold-mediated strand displacement amplification	3.1 × 10^−^^13^	[32]
surface plasmon resonance	boronic acid functionalized AuNPs	2.7 × 10^−^^13^	[33]
biofuel cells	exonuclease-powered DNA walker	4.5 × 10^−^^17^	[34]
square wave voltammetry	cascade toehold-mediated strand displacement reaction	4 × 10^−^^17^	[35]
fluorescence	dumbbell DNA switch and DSN-mediated amplification	10^−^^16^	this

## Data Availability

Data available on request from the corresponding author.
